# Telehealth and hepatitis C treatment for indigenous communities in the United States

**DOI:** 10.26633/RPSP.2020.13

**Published:** 2020-03-06

**Authors:** Jessica Leston, David Stephens, Matthew Miller, Brad Moran, Paulina Deming, Jorge Mera

**Affiliations:** Northwest Portland Area Indian Health Board Portland Northwest Portland Area Indian Health Board Portland Oregon United States Northwest Portland Area Indian Health Board Portland, Oregon, United States.; US Indian Health Service US Indian Health Service RockvilleMaryland United States US Indian Health Service, Rockville, Maryland, United States.; University of New Mexico Health Sciences Center ECHO Institute AlbuquerqueNew Mexico United States University of New Mexico Health Sciences Center, ECHO Institute, Albuquerque, New Mexico, United States.; Cherokee Nation Health Services Cherokee Nation Health Services TahlequahOklahoma United States Cherokee Nation Health Services, Tahlequah, Oklahoma, United States.

To the editor:

In the United States (US), an estimated 2.4 million persons have chronic infection with hepatitis C virus (HCV).(1) The number of deaths from HCV-related mortality is greater than that of HIV and tuberculosis combined.(2) Treatment with direct-acting antivirals (DAAs), usually 1-3 pills a day for 8 or 12 weeks, can cure over 95% of patients.(3) Successful treatment of HCV has been shown to greatly reduce liver-related as well as all-cause mortality.(4)

American Indian and Alaska Native (AI/AN) people have over twice the national rate of HCV-related mortality.(5) The largest health care provider for AI/AN communities is the Indian Health System, a national network of federal (Indian Health Service), tribal, and urban health facilities, comprised mostly of rural primary care clinics.

As part of the Indian Health System response to HCV, health facilities have access to tele-mentoring support such as the ECHO (Extension for Community Healthcare Outcomes) model, which has demonstrated excellent outcomes in treating HCV.(6) The program connects rural clinicians (‘spokes’) to a specialist team (‘hub’). These participants meet regularly via low-bandwidth video conference technology. The format of case-based learning, supported by short didactic presentations, aims to scale up clinical capacity across a health network. Patient presentations entail a brief de-identified standardized form with a patient’s clinical history to assess liver disease severity and determine optimal HCV treatment.

In the US Northwest/Northern Plains, a tribal HCV ECHO is the primary tele-mentoring option for the 65 Indian Health System facilities in Washington, Oregon, Idaho, Montana, North Dakota and South Dakota, which collectively serve approximately 554 000 members of AI/AN communities.

In 2018, the tribal ECHO program provided 165 patient consults among these six states. This region has 65 Indian Health System facilities, of which 29 (44.6%) presented an HCV patient (range 1-31 presentations, median 3.5). The same year, national Indian Health System pharmacy data documented 376 28-day prescriptions of HCV DAAs among the 65 facilities. Of these, 92.3% (347/376) DAA orders were from facilities participating in an ECHO program. Participation was significantly associated with treatment services; the majority of participating sites (24/33, 72.3%) prescribed DAAs, while only two non-participating facilities (2/32, 6.3%) did so (risk ratio 11.6, 95% confidence interval 2.9-45.6). Approximately one third (34.5%) of presenting clinicians were pharmacists, suggesting that ECHO enables case management responsibilities to be shared more widely among other members of medical teams, an important consideration in rural settings with ongoing provider shortages.

Tele-mentoring services for HCV appear to be essential to treatment at the primary care level in rural health clinics. In addition, patients, clinics and community leaders conveyed great satisfaction that HCV services are provided where patients live, from clinicians they already know, rather than requiring referral to a specialist hours away. These long-distance referrals can represent a hardship in terms of time lost, monetary cost, and being transferred to a setting with less cultural competency which can ultimately harm linkage to care. With access to highly effective HCV DAAs, tele-mentoring and ECHO is a pillar of meeting disease elimination goals in settings with limited access to specialists.

## Declaration

Authors hold sole responsibility for the views expressed in the manuscript, which may not necessarily reflect the opinion or policy of the *RPSP/PAJPH* or the Pan American Health Organization (PAHO).
